# Simultaneous Bilateral Proximal Femur Implant Failure: A Case Report

**DOI:** 10.7759/cureus.32543

**Published:** 2022-12-15

**Authors:** Smitha E Mathew, Alisa Malyavko, Sean Tabaie

**Affiliations:** 1 Orthopaedic Surgery and Sports Medicine, Children's National Hospital, Washington, DC, USA; 2 Orthopaedic Surgery, George Washington University School of Medicine and Health Sciences, Washington, DC, USA

**Keywords:** osteotomy, moebius syndrome, pediatric orthopedic surgery, implant fracture, developmental dysplasia of the hip

## Abstract

A seven-year-old boy with Moebius syndrome and bilateral hip dysplasia underwent left-sided adductor lengthening, bilateral proximal femur varus derotational osteotomies, and internal fixation with proximal femur blade plates, and left-sided Dega pelvic osteotomy. Postoperatively, he was immobilized in a Petrie cast. A month later, the child presented with bilateral proximal femur blade plate implant failure. Simultaneous bilateral proximal femur implant failure in a child, to our knowledge, has not yet been reported. Implant failure in the absence of significant trauma is rare. We describe various contributory factors that may lead to implant failure which must be carefully considered while managing a non-ambulatory child.

## Introduction

Moebius syndrome is a rare congenital neurological disease that involves several cranial nerves, largely the sixth and seventh cranial nerves [1]. In addition to cranial nerve palsies, this syndrome can also present with orofacial abnormalities, limb abnormalities, and hypotonia [2]. As a result, patients with Moebius syndrome may encounter nutritional deficiencies due to feeding difficulties as well as increased complications with airway management and general anesthesia due to oral abnormalities and hypotonia [2-3]. 

One of the more common pediatric congenital abnormalities is developmental dysplasia of the hip. Hip dysplasia can present in a variety of ways depending on the severity of the disease. Pediatric patients with hip dysplasia can present with a mildly dysplastic acetabulum or a full hip dislocation. There are both surgical and non-surgical treatment options depending on the degree of dysplasia [4]. For surgical management of hip dysplasia, orthopedic plates and screw implants are commonly used in children as internal fixation devices for the purpose of bone healing. These implants stabilize the bones while allowing adequate contact between the bone interfaces, permitting necessary compressive stresses across the fractured or osteotomized bone ends while preventing detrimental tensile stresses. This enables anatomical reduction and early mobilization [5-6]. Therefore, these implants must possess sufficient mechanical strength to overcome the physiological loading stresses during bone healing [7].

Implant failure has been mainly attributed to either significant post-surgical trauma or repeated loading of the implant prior to proper healing [8]. Additional causes include poor surgical technique, inadequate fixation, improper postoperative immobilization, and defective implant design or metallurgy [9]. In the adult literature, hardware failure is well documented, with plate failure occurring more commonly than intramedullary nail failure for long bones of the lower extremity, and often due to significant trauma. However, in pediatrics, there is a paucity of data regarding implant failure, primarily due to a child’s greater potential for bone healing and remodeling. We report a rare case of bilateral proximal femur blade plate implant failure in a seven-year-old boy with no history of postsurgical trauma and adequate postoperative immobilization. The parents provided consent for the case report.

## Case presentation

**Figure 1:**
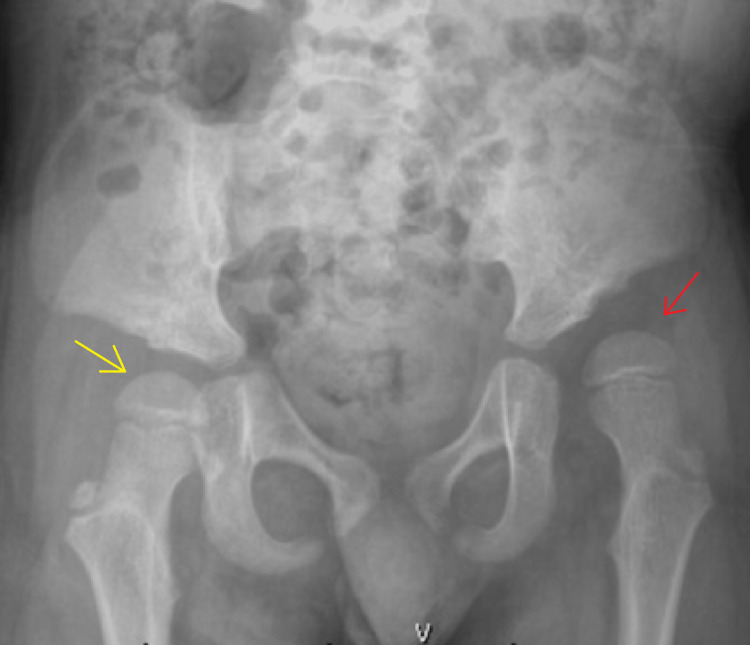
Preoperative AP radiograph of the pelvis demonstrating right sided hip subluxation with Reimer’s migration index of approximately 50% (yellow arrow) and approaching dislocation on the left (red arrow) with bilateral acetabular dysplasia.

We present a seven-year-old young boy with a history of Moebius syndrome with limited ambulation. He used a stander and occasionally a gait trainer, with no complaints of pain. On general examination, the child had poor head control but normal overall muscle tone. Musculoskeletal examination revealed a full passive range of movements of the large joints of the upper and lower extremities, except for limited left hip abduction. Pelvic radiographs revealed left acetabular dysplasia with worsening of Reimer’s migration indices bilaterally. The migration percentage was approximately 65% on the left side and approximately 50% on the right side (Figure 1).

Considering the amount of bilateral hip subluxation, acetabular dysplasia, and the patient's age, the child underwent left-sided adductor lengthening, bilateral proximal femur varus derotational osteotomies, and internal fixation with proximal femur blade plates (PediLoc® Locking Cannulated Infant Blade Plate - 90⁰ angle, 35 mm x 6 mm x 3-hole blade plate) (OrthoPediatrics, Warsaw, IN, USA) and left-sided Dega osteotomy. Postoperatively, the child was placed in a Petrie cast, with plans for cast removal at six weeks postoperatively, and subsequent progression to standing activities and physical therapy.

**Figure 2:**
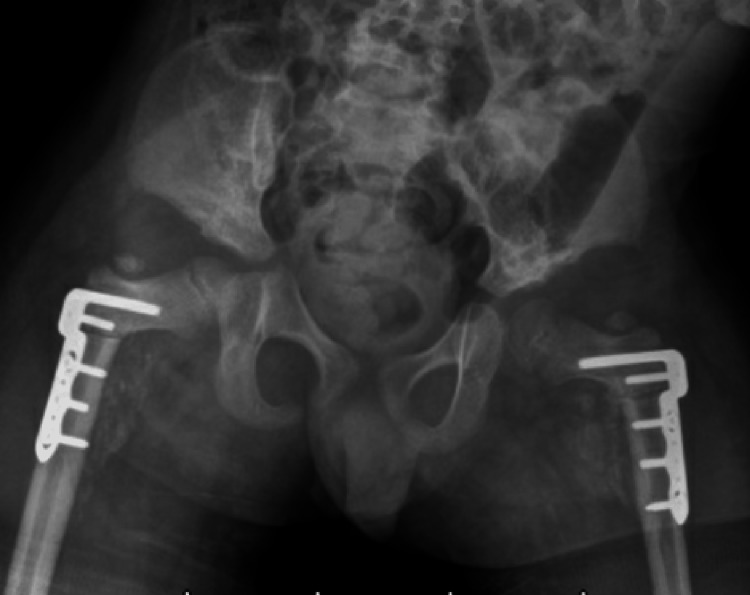
AP radiograph of the pelvis at 3 weeks follow up demonstrating bilateral well reduced hips with implants in place. There is early callus formation at the osteotomy sites.

At three-week follow-up, the child presented with a grade I pressure sore on the posterior aspect of his left ankle at the most distal aspect of the cast. He was afebrile and the pain was well controlled with non-steroidal anti-inflammatory drugs (NSAIDs) and muscle relaxants. Overall, the child remained comfortable in the cast and radiographs showed well-reduced hips with implants in place (Figure 2). The distal end of the cast was trimmed to offload the pressure sore, and the wound was managed with over-the-counter topical applicants.

**Figure 3:**
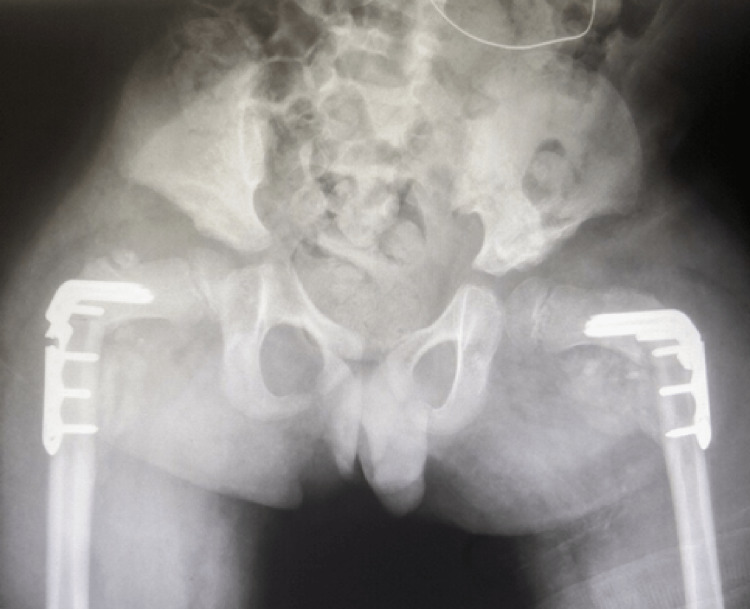
Four weeks postoperative AP radiograph of the pelvis demonstrating bilateral implant failure. AP, anterior-posterior

**Figure 4:**
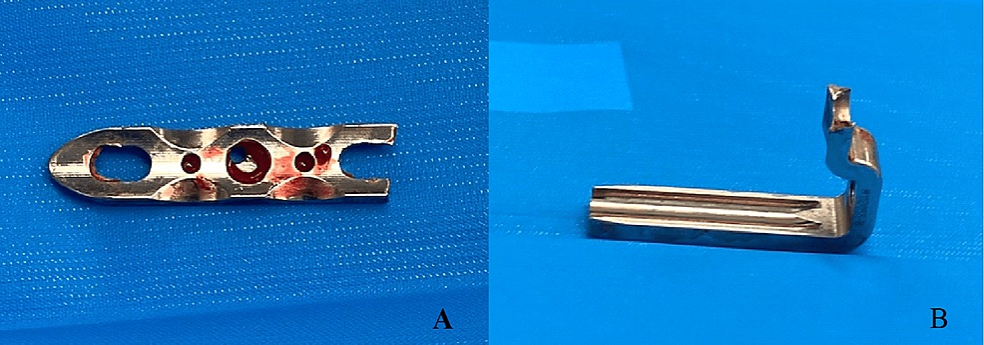
Broken implant, failed at the first non-locking hole of the side plate (same location of failure for both plates): (A) front view; (B) side view.

**Figure 5:**
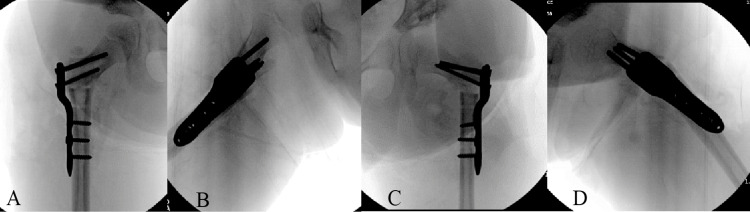
AP and lateral intra-operative views of the revised proximal fixation using proximal femoral locking plates. (A) AP right pelvis; (B) Lateral right pelvis; (C) AP left pelvis; (D) Lateral left pelvis. AP, anterior posterior

**Figure 6:**
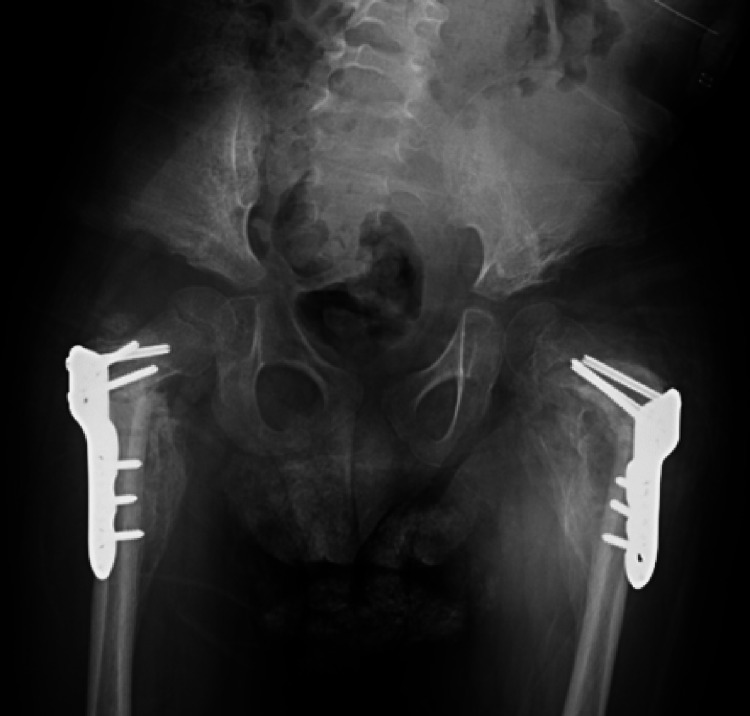
AP radiograph of the pelvis at 10 weeks post revision surgery demonstrating well-located hips, abundant callus formation at the osteotomy sites, and no evidence of implant complication. AP, anterior posterior

Five days later the child presented with complaints of loosening and slippage of the Petrie cast. Pelvic radiographs revealed bilateral implant failure of the proximal femoral locking plates, just distal to the locking screw, with loss of reduction of the proximal femoral osteotomies (Figures 3-4). He subsequently underwent implant removal and revision bilateral open reduction, realignment of varus derotational osteotomies, and internal fixation with pediatric locking compression plates (PediLoc® Locking Proximal Femur plate - 110⁰ angle, 3.5 mm x 3-hole plate) (OrthoPediatrics, Warsaw, IN, USA) (Figure 5). Following surgery, the child was placed in a double hip spica cast which was removed at six weeks postoperatively. Pelvic radiographs at cast removal showed well-reduced hips with abundant callus formation and no signs of implant failure (Figure 6).

## Discussion

Simultaneous bilateral proximal femur implant failure in a child is rare and to our knowledge has not been reported. The most common cause of implant failure in children is significant postsurgical trauma. In the absence of trauma, implant failure is rare as they are frequently removed following bone healing as retained implants have been associated with peri-implant fractures, growth disturbance, delayed/ chronic infection, metallosis, corrosion, carcinogenicity, and probable interference with future orthopedic procedures in adulthood [10-13]. Mechanical implant failure can be due to fatigue, plastic, or brittle failure. Plastic implant failure involves the deformation of the implant material whereas brittle implant failure has very little or no deformation of the material. Fatigue failure has been attributed to inferior technique, inadequate fixation and/or inadequate postoperative immobilization. In contrast, both plastic and brittle failure usually occur following mechanical overload or secondary significant trauma [9]. In comparison to intramedullary nails, orthopedic plates and screws interfere with the periosteal vascularity which could contribute to the failure of the implant [14].

In our report, the patient was diagnosed with failure of bilateral blade plates and screws at one month postoperatively, in the absence of trauma. We believe that multiple factors could have contributed to this. One contributing factor is the increased likelihood of osteopenia or osteoporosis in children who have limited mobility or are non-ambulatory due to lower bone density. Other risk factors include poor nutritional status, lack of exposure to sunlight, a low Vitamin D level, and a low calcium level. These factors can contribute to increased bone fragility with poor bone healing potential at the osteotomy site, increased risk of developing fractures, and increased likelihood of implant failure [15].

Following surgery for hip dysplasia, patients require immobilization to stabilize the hip joint in its new position. Postoperative immobilization techniques include spica, Petrie casting, or the use of an abduction pillow with bilateral knee immobilizers [16-17]. Our child was placed in a Petrie cast postoperatively. This abduction “A-frame” cast keeps the hips in the abduction and helps control pain [18]. We chose this method of immobilization as we felt the parents would not be compliant with the use of an abduction pillow with knee immobilizers. We planned on six weeks of immobilization due to poor bone quality in a non-ambulatory child.

The Petrie cast includes a wooden stick placed between the two leg casts to hold the legs in a wide-spread position. This makes it difficult to transfer the child, owing to the child’s legs being in the spread-out position, and the size and weight of the child. Hence caregivers usually hold on to the wooden stick while transferring or positioning the child, thus levering at the hips, which could increase the stresses around the osteotomies and the implants.

Infant-sized blade plates are made of stainless steel, which is stiff, dense, and has a high modulus of elasticity [19]. However, the small size of the implant and marked decrease in the cross-sectional area of plate material around the site of the proximal screw hole could contribute to decreased overall strength in comparison to larger implants used in adults, making them susceptible to failure over time. In our child, the implant failed at this region, as seen in Figure 4. Another probable cause for failure was thought to be a brittle failure. We found that both the proximal femur blade plates used in this child belonged to the same lot of implants. The failed implants were evaluated in our orthopedic research lab for their metallurgy and strength and no defect was identified, ruling out brittle failure. We are currently conducting a cadaveric biomechanical study to compare the implant metallurgy and efficacy of infant and child blade plates and proximal femur locking plates.

Following implant failure in our child, revision bilateral open reduction and proximal femoral varus derotational osteotomies with a pediatric locking compression plate followed by immobilization for six weeks in a spica cast resulted in significant callus formation and bony union at the osteotomy sites. The locking compression plate provides stability in children with poor bone density and osteoporosis and has been found to produce similar results to a pediatric proximal femur blade plate in terms of fixation and neck-shaft angle correction [20]. We opted to keep the child immobilized in a spica cast for six weeks to avoid stress on the implants and osteotomy sites. Special attention was paid to retrain the parents in care of the child while in the cast. There was also an improvement in the child’s nutritional status following the addition of vitamin D and calcium supplementation. Hence, consideration of various factors while managing a non-ambulatory child is crucial.

## Conclusions

In conclusion, we report a case of bilateral proximal femur blade plate implant failure in a child with Moebius syndrome and bilateral hip dysplasia who underwent left-sided adductor lengthening, bilateral proximal femur varus derotational osteotomies, and internal fixation with proximal femur blade plates, and left-sided Dega pelvic osteotomy. Implant failure in children in the absence of significant postsurgical trauma is rare and could be attributed to multiple causes. Special attention must be paid to improve the bone health of the child prior to surgery with Vitamin D and calcium supplementation. Postoperative management is as crucial as the surgical technique. Avoidance of stress on the operative site and the implant in the early postoperative period as well as avoidance of prolonged immobilization to prevent disuse osteopenia is essential for bone healing. Parent/caregiver education on the care of the child while in the cast is vital. Though our patient was appropriately immobilized in a Petrie cast after surgery, we believe that timely education on avoidance of levering at the hips by holding on to the wooden stick of the Petrie cast was lacking. A passive range of movements at the hips helps prevent osteopenic disuse fractures and must be encouraged. Our report also suggests that the metallurgy of pediatric implants needs to be further investigated as it can contribute to failure. Consideration of these various factors while managing a non-ambulatory child is crucial. 
