# Correction: Simultaneous Bilateral Proximal Femur Implant Failure: A Case Report

**DOI:** 10.7759/cureus.c106

**Published:** 2023-03-07

**Authors:** Smitha E Mathew, Alisa Malyavko, Sean Tabaie

**Affiliations:** 1 Orthopaedic Surgery and Sports Medicine, Children's National Hospital, Washington, DC, USA; 2 Orthopaedic Surgery, George Washington University School of Medicine and Health Sciences, Washington, DC, USA

This article has been corrected at the request of the authors to edit the name of the PediLoc® Locking Cannulated Child Blade Plate to the correct name: PediLoc® Locking Cannulated Infant Blade Plate. In addition, “Child-sized blade plates are made of stainless steel” has been changed to “Infant-sized blade plates are made of stainless steel”.

The authors deeply regret that this error was not identified and addressed prior to publication.

 

